# Domestic Filth Flies in New Haven, Connecticut: A Case Study on the Effects of Urbanization and Climate Change by Comparing Fly Populations after 78 Years

**DOI:** 10.3390/insects12110972

**Published:** 2021-10-27

**Authors:** Julie Pinto, Paola A. Magni, R. Christopher O’Brien, Ian R. Dadour

**Affiliations:** 1 Discipline of Medical, Molecular & Forensic Sciences, Murdoch University, 90 South Street, Murdoch, WA 6150, Australia; P.Magni@murdoch.edu.au; 2 Murdoch University Singapore, King’s Centre, 390 Havelock Road, Singapore 169662, Singapore; 3 Criminal Justice and Forensic Sciences Department, Henry C. Lee College of Criminal Justice and Forensic Sciences, University of New Haven, West Haven, CT 06516, USA; RcOBrien@newhaven.edu; 4 Source Certain International Pty Ltd., P.O. Box 1570, Wangara DC, WA 6947, Australia; ian.dadour@sourcecertain.com

**Keywords:** Diptera, Calliphoridae, abundance, landscape ecology, urbanization

## Abstract

**Simple Summary:**

Domestic filth fly population data were collected in the summers of 1942–1944 in the urban city of New Haven, Connecticut, during a polio epidemic. The current survey was completed 78 years later by setting out a weekly trap in the same region during June–September over a two-year period. Results indicate that the fly population has changed in the city, with 16 fewer species trapped overall, and there have been changes in the fly species trapped. Some species have increased in abundance, notably *Lucilia coeruleiviridis*, while numbers of the common *Lucilia sericata* have decreased, and *Lucilia illustris* was absent. Changes in land cover and climate were also assessed to show that the trap site has experienced significant habitat change, together with an increase in the average temperature and rainfall. Fly numbers were significantly affected by temperature and rainfall in both the 1940s and the current survey. The results of this study suggest the prolonged period of urbanization of the region is influencing the domestic filth fly population.

**Abstract:**

Changes in common and widespread insect populations such as the domestic filth fly in urban cities are useful and relevant bioindicators for overall changes in the insect biomass. The current study surveyed necrophagous flies by placing a weekly trap from June–September over a two-year period in the city of New Haven, Connecticut, to compare data on fly abundance and diversity with data collected 78 years earlier. Climate and land cover changes were also assessed in combination with the fly population for each period. The survey results suggest the domestic filth fly population is now less diverse with decreased species richness and changes in the relative abundance of species. In both surveys, 95–96% of the population was composed of only three species. The current survey data indicate the numerical dominance of *Lucilia sericata* has decreased, the abundance of several species, notably *Lucilia coeruleiviridis*, has increased, and *Lucilia illustris* is absent. Species that showed a significant interaction with temperature in the 1940s survey have now increased in abundance, with several of the trapped species continuing to show an interaction with temperature and rainfall. Analysis of the land cover and climate data characterizes the trap site as a region exposed to a prolonged period of industrialization and urbanization, with only 7% of the land cover remaining undeveloped and over 50% impervious, coupled with an increase in temperature and rainfall. This study serves as a model for changes in domestic filth fly populations and other insects in similarly highly urbanized established cities.

## 1. Introduction

The poliomyelitis epidemic in the United States prompted the study of local fly populations in New Haven, Connecticut, in the early 1940s [1,2]. Over three successive years, 1942–1944, flies were trapped weekly from May to October in New Haven after reports of poliovirus isolation from several fly genera and species [3,4,5]. The aim was to obtain information about the fly population and to find possible seasonal correlations with the total population, individual species, and the polio epidemic. Fly traps were placed near the Yale New Haven Hospital (41°18′12.2″ N 72°56′08.1″ W) located near the “Hill” region, which was the original focus of the 1943 poliomyelitis epidemic in the city. Flies are responsible (not solely) for the transmission of a variety of viral, bacterial, and protozoal pathogens of public health importance [6,7,8,9,10] with domestic families of filth flies including flesh flies (Diptera: Sarcophagidae), house flies (Diptera: Muscidae), and blow flies (Diptera: Calliphoridae) acting as vectors of human disease. Transmission is facilitated by their synanthropic feeding and reproductive habits and their ability to mechanically transmit disease by the translocation of pathogens through defecation, regurgitation, and on their exoskeleton [6,11].

The historical collection of local population data offers the rare opportunity to compare the abundance and composition of domestic filth fly species of a specific locality to present day samples. In addition to their medical significance, flies are also forensically and ecologically important. The forensic application of blow flies to death investigations is now also recognized and has been extensively used since the 1980s [12,13]. Blow flies are the primary colonizers of decomposing bodies, making their species-specific developmental data together with the local temperature of value when estimating the minimum postmortem interval [13,14].

The ecological importance of blow flies as necrophagous consumers, decomposers, parasites, and as pollinators [15,16] is fundamental to the ecosystem. Blow flies have adapted to live in highly modified environments that are often inhospitable to other insects [17], resulting in their common and wide distribution. In the past, the abundance of common and widespread species has been overlooked, and species-level conservation has given priority to rare species at risk of imminent extinction [18]. It has become increasingly apparent, however, that common and widespread species with strong anthropogenic ties have not been immune to declines in abundance and extinction, and the effects are having profound consequences on the ecosystem [19,20,21].

Common species numbers have been found to disproportionately impact a large number of species and geographical areas with even relatively small reductions in abundance [22]. Blow flies have been considered poor ecological indicators due to their status as common, widespread, and highly mobile generalists lacking established correlations with ecosystem change [23,24]. It is, however, the common and widespread distribution of blow flies and domestic filth flies, together with their taxonomic diversity, rapid fluctuations in population density, and presence in anthropogenic environments [25,26], that makes these species ideal for monitoring ecological changes created by environmental degradation and climatic change.

A global decline in insect abundance and species richness has been observed since the start of the 1900s, followed by a further decline during the 1950s and 1960s and an increasing decline in the last two decades [27]. A current study compiling data from 166 long-term surveys of insect assemblages showed that the strongest evidence for declines in terrestrial insect assemblages was found in North America [28]. Major insect taxonomic orders have seen a decline in species numbers, with some populations, mainly agricultural herbivores and nuisance pests, increasing [29]. A decline of 1% to 2% per annum is frequently reported, especially in areas affected by human activity [30]. This decline has been attributed to habitat loss, pollution, biological factors such as pathogens and introduced species, and climate change. Habitat loss in its many forms including deforestation, fragmentation, urbanization, and agricultural conversion is seen as a primary driver [30].

A decline in insect species is often accompanied by the presence of invasive species [31]. Blow fly range expansion has been noted for some time [32], with multiple recent examples, and thought largely attributable to climate change and globalization [33]. *Lucilia cuprina* Wiedemann, indigenous to Africa and Asia, is now found throughout the warm regions of the world. It has recently been recorded as far north as Indiana in the US, along with *Chrysomya megacephala* (Fabricius), another invasive species originally from Australasia [34]. *Chrysomya rufifacies* (Macquart), a native of Australasia, was first detected in Central America in 1978, and has since spread into the southern US in 1981 [35] and Ontario in 2004 [36]. This trend in range expansion is likely to increase in incidence with the continued change of urbanization and climate.

Changes in insect abundance and species richness in the larger region encompassing the site of the current and 1940s surveys have become increasing apparent in recent decades. A marked and ongoing decline of many moth and butterfly species has been recorded in Connecticut and neighboring states, including some previously common species and populations in habitats that remain rural, experiencing little difference in urbanization or agricultural practices [37]. The establishment of some southern species and the increase in abundance of previously rare species have also been documented, possibly attributable to a longer growing season and warmer summer temperatures [37]. Likewise, the abundance of mosquitoes in Connecticut has increased by close to 60% and species richness by 10% since 2001, with the total species richness highest in the southern portion of CT including New Haven, due to the northward range expansion of multiple species within the *Aedes, Anopheles, Culex*, and *Psorophora* genera (Diptera: Culicidae) [38]. Changes to flora have accompanied these changes in the insect population, with the northeastern United States now having the highest proportion of nonnative species in the continental United States [39].

At the time of the New Haven 1942–1944 survey, the authors reported that very few filth fly data existed for the northeastern United States. Today, this remains the case [40]. More data are needed to determine if the biodiversity decline seen in many of the major insect taxa is also being witnessed in Diptera. Data re lacking for most Diptera [27], with data only available on muscid flies [41], mosquitoes [38], and other flies including hoverflies [29]. The proximity of the current and historical survey sites enables the assessment of attributing factors such as land use change and climate and serves as a model for similarly highly urbanized established cities such as New Haven. This study compares current fly data to the data collected in 1942–1944 to assess changes in species diversity and relative abundance. Changes in land cover and climate were also assessed by examining weather data from the last 78 years and comparing present day land use to historical data to determine the effect if any of the environment. The current survey provides a much needed comparison to the fly data collected 78 years ago.

## 2. Materials and Methods

A hanging Diptera cone trap [42] baited with 200 g of raw pork meat was placed weekly from 9 a.m. to 4 p.m. from the beginning of June to the end of September in 2019 and 2020. The trap was hung approximately 1.5 m from the ground in an overgrown partly shaded lot between two buildings. The trap set out at the end of September 2020 was placed adjacent to a pig head to maximize collection for that time point. The fly population data collected were compared to the results of a study conducted in 1942–1944 approximately 2 km from the present day trap site. As species counts were not published for 1942, the 1943–1944 fly counts were used to compare fly populations across the two studies. In 1943–1944 a conical hoop flytrap [1,43] was placed twice a week and baited with a mix of feces and pooled mouse tissue. In the current study, calliphorids were identified using Whitworth’s updated key [44], and non-calliphorids were identified using Dodge’s 1953 key [45].

ArcGIS [46] was used to define current land cover by using a 5 km buffer projected out from the Yale New Haven Hospital, which encompassed the 1940s and current trap sites, and adding the 2016 National Land Cover Database (NLCD), the 2016 NLCD urban impervious surfaces data, and the Trust for Public Land urban heat island data. The NLCD land cover data include 19 categories of land use at 30 m resolution, and NLCD Urban imperviousness data represent the percentage of urban impervious surfaces such as roads and core urban areas over every 30 m pixel. The urban heat island data were generated from ground-level thermal sensors from the 2018 and 2019 summers and show the heat severity for every 30 m pixel using a 1 to 5 scale, with 5 being a heat area significantly above the mean for the city. Land cover changes were assessed using a 1954 USDA land cover map [47], 1985 land cover data from the UConn Centre for Land Use Education and Research (CLEAR), and USGS topographic maps from 1892, 1942, and 1954.

The city of New Haven is a coastal city located on the North-East Coast of the United States that has undergone significant changes in the landscape since the comparative fly survey conducted in the early 1940s. By the turn of the 20th century, New Haven was already a major industrial hub with a population of over 100,000. At the time of the 1940s fly survey, the city was a dense urban setting at its industrial peak with a population of approximately 160,000. In the 1950s, it underwent major urban renewal after the loss of population and industry. Large sections of the city were redeveloped, major highways were constructed, and residential housing booms occurred in adjacent suburban towns [48]. Today, New Haven is completely built out, except for sections of land designated for conservation such as parks and open space. The city is less dense and industrial, with a population of approximately 130,000 (US Census).

New Haven typically experiences a large range in temperature, both daily and annually, with January being the coldest month with a mean temperature of 0 °C and July being the hottest month with a mean temperature of 23 °C. Precipitation is normally evenly distributed throughout the year, with June being the wettest month with a mean rainfall of 26 mm, and February the driest with an average of 14 mm (noaa.gov). Summers are now considered hotter and drier, and spring is arriving earlier with increased precipitation and heavier rainstorms [49].

### Statistical Analysis

Temperature and rainfall data for the current 2019–2020 survey years were obtained from the NOAA Tweed Airport-New Haven weather station located approximately 7 km from the trap site. Weather data from the nearby city of Bridgeport, Connecticut, were obtained for 1942–1944 to analyze fly collection data from those years. While collection dates for the 1942–1944 data were not published, fly species counts were obtained from the published graphs, temperature data were averaged biweekly, and rainfall data totaled biweekly, to compare the historical fly collection data to temperature and rainfall data. A one-way analysis of variance (ANOVA) was used to determine if a statistically significant interaction between fly species counts and the maximum temperature four weeks prior to and on the day of trapping and rainfall four weeks prior to and on the day of trapping for 1942–1944 and 2019–2020.

Furthermore, to determine if species fly counts were significantly different across the two surveys and between years of each survey, z scores and *p* values were computed. The Simpson’s diversity index was used to measure the diversity of the fly population for both surveys, considering the number of species present as well as the relative abundance of each species. Rank distribution graphs were plotted to visualize species evenness.

## 3. Results

A total of 1352 flies were trapped across the two years, with the highest number of flies peaking on both years between the middle of June and early July. The number of species was highest in July in 2019 and in June and September in 2020. A total of 14 species were trapped with the number of species not exceeding 10 in any one month. Ninety-five percent of the total overall count comprised of three species: *Lucilia sericata* (Meigen) 56.76%, *Lucilia coeruleiviridis* Macquart 25.41%, and *Phormia regina* (Meigen) 13.37%. The three dominant species remained the same across the two years (Table 1). In 2019, *L. sericata* accounted for 35.97% of the total count, *L. coeruleiviridis* 39.68%, and *P. regina* 18.71%, and in 2020, *L. sericata* accounted for 74.52% of the total count, *L. coeruleiviridis* 13.22%, and *P. regina* 8.82%, while all other species accounted for 1% or lower. Flies were also ranked according to an arbitrary number according to their abundance in each trap, and the top three species according to the overall arbitrary rank was the same as their rank according to overall count.

Ninety-six percent of the total overall count in 1943–1944 also comprised only three species: *L. sericata*, 83.75%; *Lucilia illustris* (Meigen), 7.48%; and *P. regina*, 5.19%, with *L. sericata* being the species with the highest number in 100% of the traps. In the current study, *L.sericata* remained the species with the highest overall trap number; however, *L.sericata* was trapped significantly less (*p* < 0.001), accounting for only 56.76% of the overall numbers, and was the species with the highest number in 59% of the traps. In the current survey, *L. coeruleiviridis* and *P. regina* counts were significantly higher in the current survey (*p* < 0.001), with *L. coeruleiviridis* surpassing *L. sericata* as the highest trapped species in 41% of the traps and *P. regina* surpassing *L. sericata* as the highest trapped species in 11% of the traps. *L.coeruleiviridis* accounted for 25.41% of all flies in the current survey, while it was relatively rare in 1943–1944, accounting for only 0.39% of the total count. The numbers of *Calliphora vicina* (Robineau-Desvoidy), *Hylemya* spp., and *Hydrotaea ignava* specimens were also significantly higher in the current survey than the numbers trapped in the 1943–1944 traps (*p* < 0.01). In the 1940s, *L. illustris* was the second-highest species trapped; however, it was not trapped in the current survey (*p* < 0.001). The number of *Sarcophaga* spp. specimens trapped in the current survey was also significantly lower (*p* < 0.001) in the current survey (Table 2, Table 3 and Table 4).

**Figure 1:**
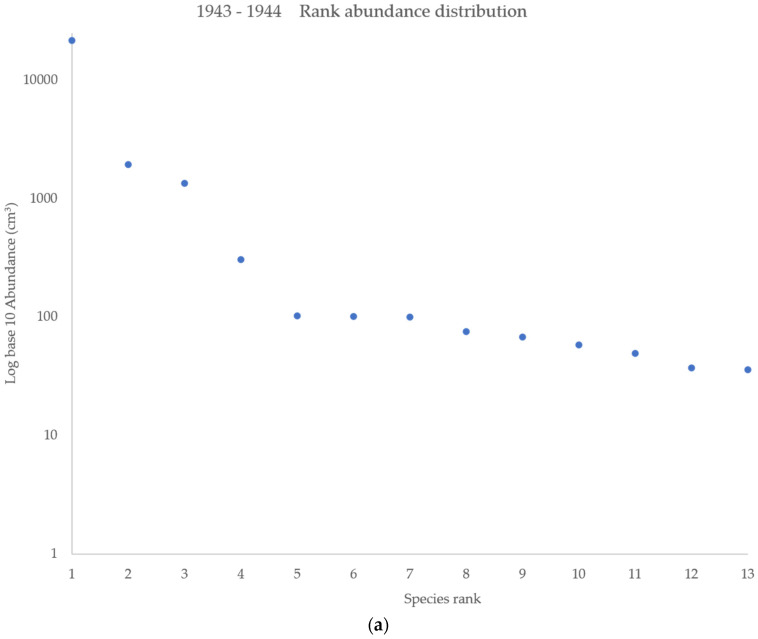

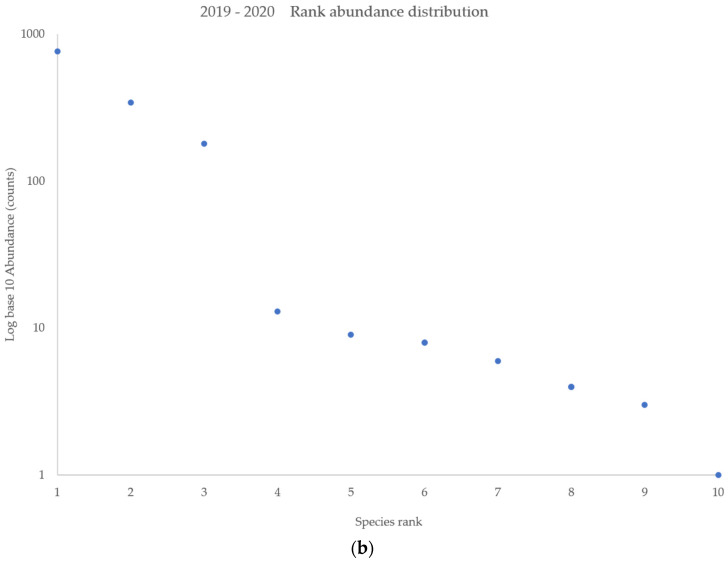
Rank abundance distributions for the (**a**) 1943–1944 survey and (**b**) 2019–2020 survey. Steep gradients are observed in both the 1940s and the present-day surveys, indicating low species evenness due to the much higher abundances of species ranked 1–4 in the 1940s and species ranked 1–3 in the present-day survey.

Sixteen additional species were trapped in the 1940s survey compared to the current survey. All of the species trapped in the current survey were also trapped in the 1940s survey, except for one species, *Calliphora livida* Hall. Diversity was higher in the 1943–1944 survey compared to the current survey. The Simpson’s diversity index showed higher diversity in 1943–1944 (D = 0.7098) compared to 2019–2020 (D = 0.4044). Rank abundance distribution graphs show low species evenness across both surveys as a result of the higher abundance of the three to four top-ranking species (Figure 1).

Fly populations fluctuated markedly from year to year in the 1940s survey and in the current survey. Significantly higher numbers of *L. sericata* were trapped in 2019 compared to 2020 (z = 14.23, *p* < 0.001), and significantly lower numbers of *P. regina* (z = −5.31, *p* < 0.001) and *L. coeruleiviridis* (z = −11.11, *p* < 0.001) were trapped in 2019 compared to 2020. In the 1940s surveys, *L. sericata* was trapped in high numbers across the three years, while *P. regina* and *L. illustris* numbers fluctuated greatly (Table 3).

**Figure 2:**
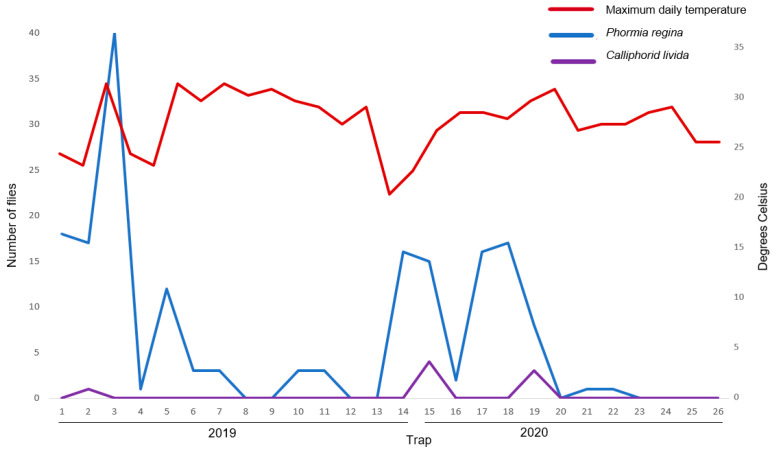
Maximum daily temperature and *Phormia regina* and *Calliphora livida* numbers for 2019–2020 traps.

**Figure 3:**
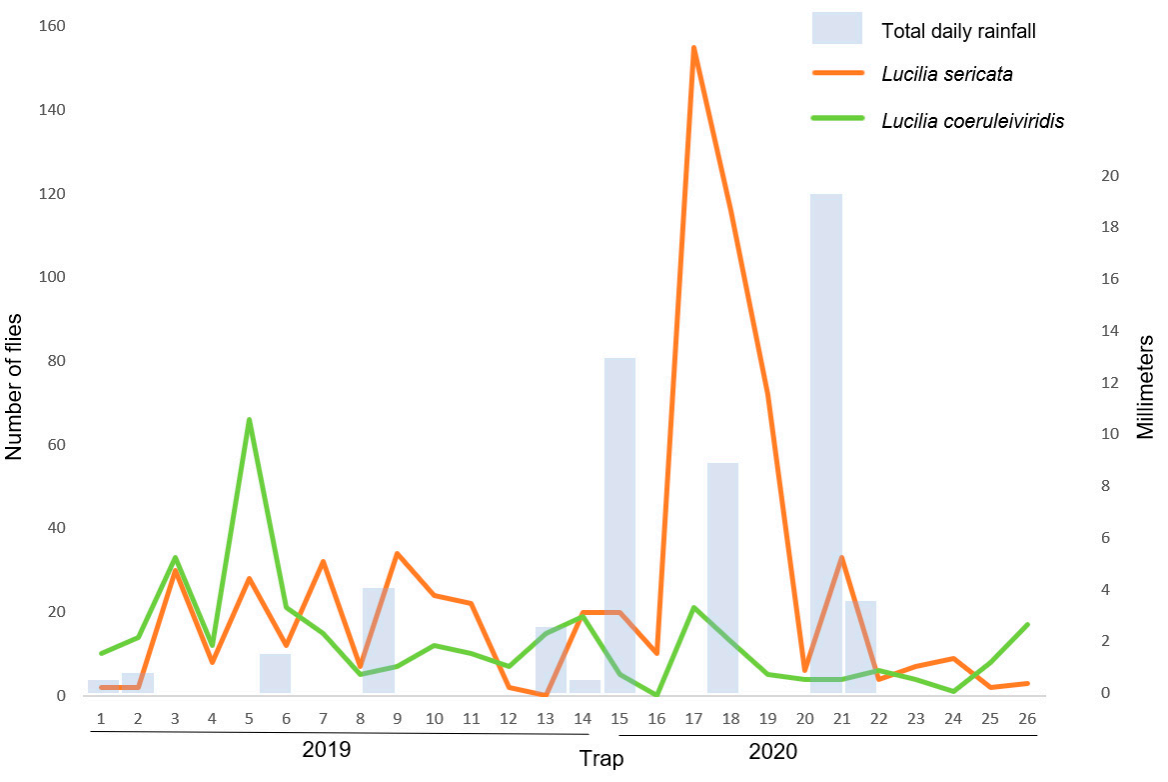
Total daily rainfall and *Lucilia sericata* and *Lucilia coeruleiviridis* numbers for 2019–2020 traps.

In the current survey, the maximum daily temperature four weeks prior to the trap date had a significant effect on *P. regina* and *C. livida* numbers, with an increase in temperature resulting in a significant increase in *P. regina* (F(_11,15_) = 11.34, *p* < 0.001) and *C. livida* (F(_3,23_) = 13.08, *p* < 0.001) counts (Figure 2). The rainfall four weeks prior to the trap date had a significant effect on *L. sericata* and *L. coeruleiviridis* numbers, with an increase in rainfall resulting in a significant increase in *L. sericata* (F(_21,5_) = 14.55, *p* < 0.01) and *L. coeruleiviridis* (F(_16,10_) = 4.43, *p* = 0.01) counts (Figure 3). No correlation was observed between temperature and rainfall for the remaining trapped species.

Temperature and precipitation also influenced *P. regina*, *L. coeruleiviridis*, and *C. vicina* counts in 1942–1944. The maximum daily temperature had a significant effect on *L. coeruleiviridis* counts on the trap day (F(_13,136_) = 2.42, *p* < 0.01) and four weeks prior to the trap day (F(_13,136_) = 5.46, *p* < 0.001), on *P. regina* counts on the trap day (F(_21,128_) = 3.89, *p* < 0.001), and four weeks prior to the trap day (F(_21,128_) = 2.67, *p* < 0.001), and *C. vicina* counts on the trap day (F(_20,142_) = 2.87, *p* < 0.001). The rainfall four weeks prior to the trap date also had a significant effect on *L. coeruleiviridis* numbers (F(_13,136_) = 2.53, *p* < 0.01) and *C. vicina* numbers (F(_20,142_) = 2.12, *p* < 0.01). No interaction between temperature and rainfall was observed for *L. sericata* and *L. illustris* 1942–1944 trap numbers.

**Figure 4:**
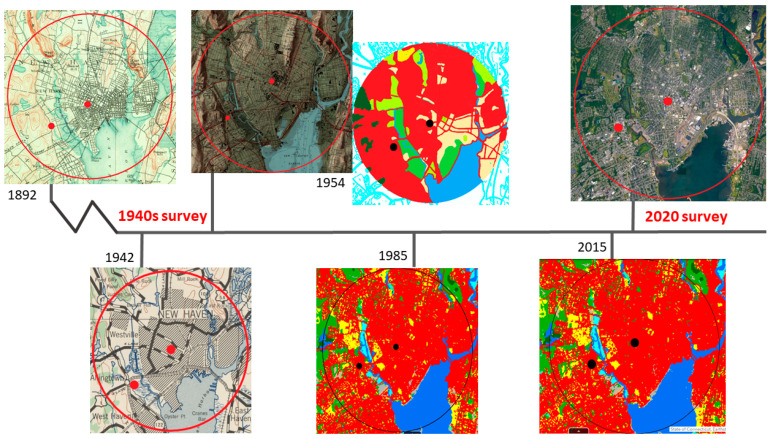
Topography and land cover of New Haven, CT, from 1892–2015. Source: 1892 [51]; 1942 [52]; 1954 [53]; 1954 land cover [47]; 1985 and 2015 land cover [50]; 2020 [54].

**Figure 5:**
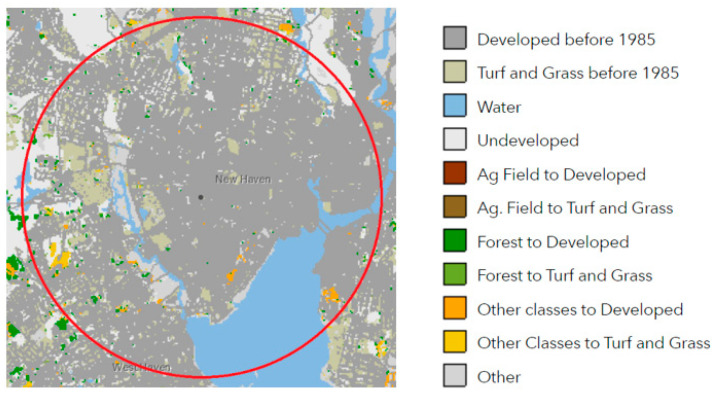
Land cover change 1985 to 2015 (UConn CLEAR).

The USGS topography map of New Haven from 1892 shows that New Haven was already an established city by 1900. The USGS topography map of New Haven from 1954 shows the original coastline, and the 1954 land cover map shows the majority of land in the 5 km buffered area as developed and a change in coastline. Comparative land cover data from 1985 to 2015 show that the changes in landcover have been minimal in this 30 year period, with only small areas of forest changing to developed and other classes changing to turf and grass (Figure 4). Land cover has changed little across the state of Connecticut from 1985 to 2015 with a 3.1% increase in developed land, a 1.6% increase in turf and grass, a 3.5% decrease in forested land, and a 1.4% decrease in agricultural fields. In this 30 year period, New Haven has gained 275 acres of developed land and lost 152 acres of forested land [50] (Figure 5).

**Figure 6:**
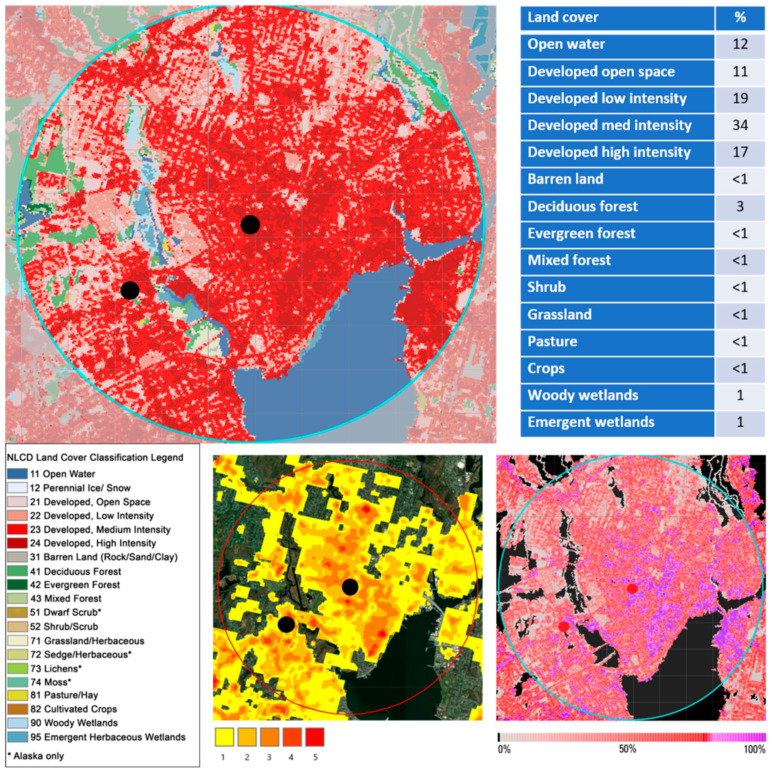
Current land cover characterization of 5 km buffered area around trap sites for current survey (left marker) and 1940s survey (right marker). Clockwise: Land cover 2016 (NLCD); urban impervious surfaces 2016 (NLCD); urban heat island severity 2019–2020 (The Trust for Public Land). The urban heat island scale 1 to 5 scale is described as 1: heat area slightly above the mean for the city and 5: heat area significantly above the mean for the city.

Present-day land cover analysis described the land in the 5 km buffer as 81% developed, 12% open water, and 3% deciduous forest, with all other land cover categories at 1% or less, and 51% of the developed surfaces having an imperviousness percentage above 50% (68% above 25% and 22% above 75%). Heat island data categorized over half of the buffered area as slightly hotter than the mean for the city, with multiple spots categorized as significantly higher (Figure 6). Tree canopy percentage is low at 16% at the original trap location [55].

## 4. Discussion

Results of this two-year study indicate the domestic filth fly population has changed in New Haven in the last 78 years. The population is less diverse with decreased species richness and changes in the relative abundance of species. The biggest change in the fly population compared to 78 years ago includes the inclusion of less species overall, the decreased numerical dominance of *L. sericata*, the increased abundance of *L. coeruleiviridis*, and the absence of *L. illustris*. Low species evenness was observed across both surveys due to the relatively much higher abundance of the highest-ranked species. In the 1940s, the numerical dominance of *L. sericata*, and also the relatively higher abundance of *P. regina*, *L. illustris*, and *Sarcophaga* species compared to all other trapped species, reduced species evenness. Species evenness was also reduced in the present-day survey as a result of the disproportionately higher abundance of the top three species: *L. sericata, L. coeruleiviridis*, and *P. regina.*

At the time of the 1940s survey, *L. sericata* had not yet achieved cosmopolitan status due to its absence in regions including Central and South America. Today, this common fly is distributed worldwide [56,57] with varying degrees of synanthropy [58,59] and often abandons its natural rural habitat in favor of human settlements especially in colder regions [8,60,61,62]. *Lucilia sericata* has adapted by being generalistic and opportunistic, feeding on a wide variety of food substrates, and being a facultative parasite, notorious for causing sheep myiasis [63]. *Lucilia sericata* remained the most abundant species trapped overall; however, it was not always the most abundant species in weekly traps, as reported in the 1940s. Temperature did not significantly affect *L. sericata* numbers in the current study nor the 1940s survey, while rainfall was a factor in the current survey. Four weeks prior to the trap date spans the entire lifecycle of the trapped specimens and coincides with the approximate time of oviposition [26]. The rainfall four weeks prior to the trap date had a significant positive effect on *L. sericata* numbers. This finding is supported by research showing *L. sericata* colonizes carcasses in water (along with only *Cochliomyia macellaria* and *Sarcophaga bullata*) [64], and the species’ ability to survive submerged in water as pupae is longer than *C. vicina* [65] and *C. vomitora* [66]. *Lucilia sericata* numbers fluctuated greatly between the first and second year of the current survey with the second year showing significantly higher numbers of *L. sericata*, coinciding with a much wetter year.

*Lucilia coeruleiviridis* was rarely trapped in the 1940s survey and in the 1950s in New York [67] while it was the second highest trapped species in the current survey. Distribution of the species is throughout the Nearctic region [68,69] and has recently expanded into Canada [68,70]. The species was previously listed as common in the southwestern US [56], while it is now a dominant blow fly species on the US East Coast, especially in the summer, as reported in Virginia [71], Florida [72], and New Jersey [73], where it is often reported as the most abundant species, the first to arrive on carrion, and the first to complete development [72]. High numbers of the species, second only to *L. sericata*, were also trapped in Hartford County, approximately 65 km north of the New Haven trap site [42]. Temperature and rainfall both significantly affected *L. coeruleiviridis* numbers in the 1940s survey. In the current survey, temperature did not have a significant effect on *L. coeruleiviridis* numbers, while rainfall four weeks prior to the trap date, had a significant positive effect, as with *L. sericata*. However, unlike *L. sericata, L. coeruleiviridis* had significantly lower numbers in the second (wetter) year of the current survey, suggesting other factors may have limited *L. coeruleiviridis* numbers in the second year, such as the high abundance of *L. sericata*, a species with a very similar developmental time. Research has shown *L. coeruleiviridis* to oviposit on carcasses in water at higher numbers than *P. regina* [74]; however, no data could be found comparing *L. coeruleiviridis* and *L. sericata*. Analysis of the 1942–1944 data showed that *L. coeruleviridis* numbers were also significantly affected by rainfall four weeks prior to the trap date, as were the maximum daily temperature on the trap date and four weeks prior, unlike *L. sericata*.

*Phormia regina* is found throughout most of the northern continents of the world, [75], is a secondary myiasis producer, and one of the primary species used to indicate the postmortem interval in human deaths throughout North America [76]. *Phormia regina* was the third highest trapped species in both the 1940s survey and in the current survey, with percentages significantly higher in the current study. The maximum temperature four weeks prior to the trap date had a significant effect on *P. regina* numbers, with an increase in temperature resulting in a significant increase in numbers. Analysis of the 1942–1944 data also showed that the temperature four weeks prior to the trap date, as well as the temperature on the trap date, significantly affected *P. regina* numbers. The species is typically considered a cold weather fly throughout most of the United States but has been recorded in large numbers during the summer in Virginia [71], Kansas [77], and California [78]. *Phormia regina* numbers also fluctuated significantly between Years 1 and 2 of the current survey, with numbers lower in the second year when *L. sericata* numbers were greater. Past research has shown the interaction between *P. regina* and *L. sericata* to be either competitive or facilitative, depending on temperature and larvae stage [79].

*Lucilia illustris* was the second most abundant fly in the 1940s survey and the most abundant fly together with *L. sericata* trapped in 1959–1960 in New Haven [80]. This was in sharp contrast to numbers captured in New York City in 1953 where the species comprised of only 1% of the total trapped [67]. *Lucilia illustris* was absent from the current New Haven traps. In nearby surveys, the species has only been trapped in very low numbers in Hartford county in 2019 and not in 2020 [42] and in very low numbers in New Jersey in 2011–2013 but not in 2014 [81]. The species is still currently one of the most abundant necrophagous Diptera in areas such as New Brunswick, Canada, together with *P. regina* [82] and was found to be the numerically dominant species in southern Finland for 15 years [83].

*Lucilia illustris* is found throughout the Holarctic and Oriental regions [68] and is responsible for secondary facultative myasis in livestock [84]. The species exhibits varying degrees of synanthropy, depending on the region. In Finland, *L. illustris* demonstrates no synanthropy [85], while in Central London, it shows a preference for urban settings [86]. Research on the competition of fly species in southern Finland showed that the survival of *L. illustris* declined even at low densities compared to other species, possibly related to the observed voracious appetite of *L. illustris* [83]. Similar findings were found in New Brunswick, Canada, with *L. illustris* overrepresented in small bait traps compared to carcasses, thought to be the result of the species’ inability to compete with the dominant species on carcasses [82]. While it is difficult to say with certainty why the number of *L. illustris* trapped has declined in the New Haven region and nearby areas, the substantial change in abundance apparent between the surveys completed in the 1940s to that completed in New York City a decade later and the current survey suggest that the species’ numbers have declined as a result of urbanization.

*Calliphora livida* was not trapped in the 1940s survey, while it was trapped in the current survey. *Calliphora viridescens* Robineau-Desvoidy, thought to be the same species at the time, was also not trapped. *Calliphora livida* is found throughout the Nearctic region [68,69] and was caught in high numbers in the late 1940s and 1950s during the first week of May in Ontario, Canada, and in the US states Georgia and Wisconsin [87]. Given that the species was only first described by Hall in 1948, it is possible that the species was misidentified as *C. vicina*, a similar species, in the 1940s surveys. The number of *C. vicina* specimens trapped were significantly higher in the current survey than the number trapped in the 1943–1944 traps. Analysis of the 1942–1944 data also showed that the temperature on the trap date and the rainfall four weeks prior had a significant effect on *C. vicina* numbers. *Calliphora vicina* is a cosmopolitan species closely associated with humans [58] and is ranked as the heaviest potential sanitary risk in Argentina along with *L. sericata* on Mihalyi’s danger index, which takes into account the fly’s habits in relation to infection transmission and the size of the fly [88].

Characterization of land cover in the 5 km buffer surrounding the trap sites has demonstrated that almost all the land in this area is now developed, with only 7% of the land undeveloped. Over half of these developed areas in the buffered area are impervious. Impervious surfaces are defined as any material that prevents the infiltration of water into the soil [89]. Such surfaces are considered a key environmental indicator as they are linked to detriments beyond the immediate habitat loss, as they prevent natural pollutant processing in the soil, transport pollutants into the waterways, and are linked to an increase in ambient temperature, soil compaction, and soil and air pollution [89,90]. Areas with larger areas of impervious surfaces are found to have fewer flying arthropods overall, especially Hemiptera, Araneae, and Diptera [91], and are found to be the key factor affecting bee assemblages [90].

Most of the land cover changes that took place in the surveyed area took place long before the comparative 1940s survey, with the region having a long history of anthropogenic change coinciding with the length of time since European settlement. A decade before the 1940s survey, rivers in the 5 km buffered area were dredged, and considerable areas of salt marshes were filled in. Changes that occurred since the 1940s survey are also significant, especially major changes to the coastline. During the 1950s and 1960s, tidal wetlands were filled for the construction of a interstate highway, dramatically changing the coastline, and by 1970, 60% of tidal wetlands had been destroyed [92]. Such changes have created opportunity for some species but may have been the tipping point for several other species already stressed in an environment subjected to a prolonged period of industrialization and urbanization.

**Figure 7:**
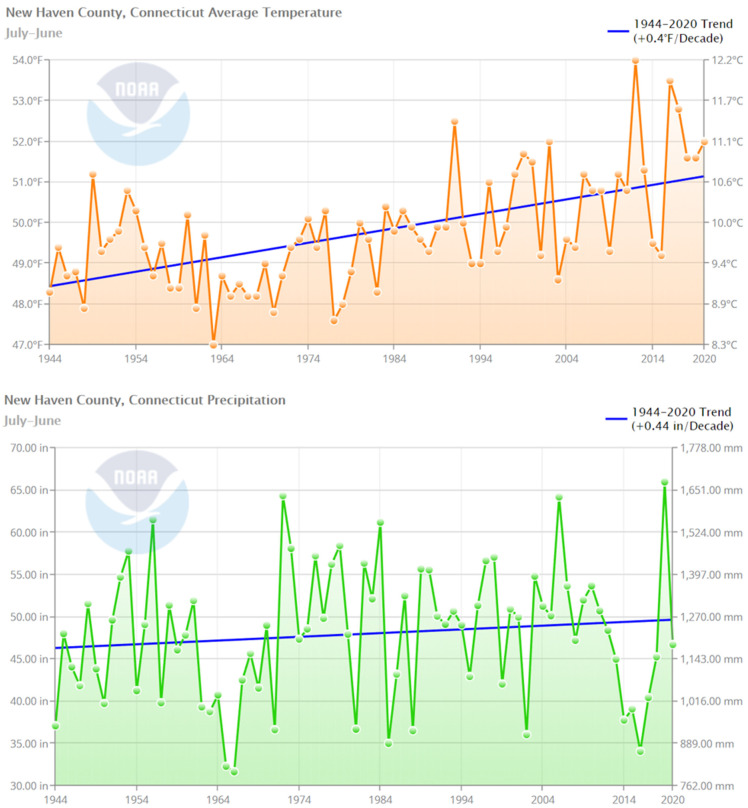
Average temperature (**a**) and precipitation (**b**) in New Haven County, Connecticut, from 1944 to 2020 (NOAA).

Since the 1940s survey, temperature, precipitation, and the number of heavy rainfall events have increased in the trapped region. Since 1944, the average temperature in New Haven has increased by 1.54 degrees Celsius, and average precipitation has increased by 78 mm (Figure 7) [93]. From 1960 to 2019, the annual number of heavy rainfall events (three consecutive days with cumulative precipitation of three inches or more) significantly increased [94]. Like land cover changes, climatic changes were also evident before the 1940s survey, with the mean temperature for the years 1907–1948 recorded at 1.3 degrees Celsius warmer than the 1780–1822 mean. Flies are likely to be among the species that respond positively to a warmer climate, with even small increases in temperature resulting in large increases in fly population density [26]. Temperature has been shown to be the primary weather variable that affects trap numbers of *L. cuprina* [95]. Overall percentages of the numbers of *P. regina, C. vicina, L. coeruleiviridis*, and possibly *C. livida* all increased since the 1940s survey, suggesting that the heat-tolerance for these species has increased, whereas *L. sericata, L.illustris*, and *Sarcophaga* species, along with several other less-trapped species, have decreased in abundance. It is interesting to note that the species that increased in abundance since the 1940s (*P.regina, C. vicina* and *L. coeruleiviridis*) were also the species that showed a significant interaction with temperature in the 1940s, while the species that showed a decrease in abundance (*L. sericata* and *L. illustris*) did not show an interaction with temperature in the 1940s. It is difficult to assess whether such changes in abundance are the result of weather changes due to the lack of additional timepoints between the two surveys and due to the correlation between climate, land use changes, and human behavior that in turn feed back into climate change [96]. Temperature increase is exacerbated in urban areas due to heat islands resulting from a combination of impervious surfaces and human activity, resulting in pockets of heat often as much as 12 °C hotter than adjacent areas [97]. The buffered trap area includes many heat islands including several of Level 5 severity, exposing species within the area to temperature increases higher than surrounding areas.

Urbanization has drastic and increasingly widespread effects, replacing natural systems with smaller sealed fragments of habitat [98]. Key factors such as local climate and nutrient availability are changed, and the structure of ecological communities is altered [99]. As urbanization exposes organisms to these novel environmental challenges, species richness and diversity decline [100,101] and biotic homogenization increases [98]. Differences in the competitive abilities of fly species when coexisting in these patchy resources needed for food and oviposition lead to the ecological displacement and local extinction of some and the prevalence of others [102,103]. This loss of diversity in developed areas has been demonstrated in other fly communities, with calliphorids found to be more diverse in natural areas compared to artificial clearings in Brazilian Amazon [25,104], Argentina [105], and across Canada [106], while an increase of abundance has been observed in sarcophagids in cleared land [25]. Biotic homogenization was already apparent in the 1940s, with *L. sericata* accounting for 80 and 90% of the volume of all flies trapped, and continues today, driven by the hyper-proliferation of a limited number of species in the population. In the 1940s, 96% of the total number of flies was represented by three species, and this trend continues today, with 95% of the total represented by three species. While *L. sericata* remains an abundant species in the region, *P. regina* and especially *L. coeruleiviridis* have increased in abundance, while *L. illustris*, previously the second-most-abundant species, now appears to be absent.

The concept of winners and losers in the face of globalization and climate change is widely accepted, with the characterization of the winners as exotic species and the losers as native. However, this is often not an accurate portrayal, as biotic homogenization also occurs without the invasion of alien species, and native biotic assemblages are often rearranged when subjected to anthropogenic disturbance [107]. All species that differed in abundance between the 1940s and the current survey, including *L. sericata, P. regina, L. coeruleiviridis, L. illustris, C. vicina*, and *C. livida*, are native to the region [68,69] and should be considered as such given their length of time in the region and evidence in the current environment of life history traits being as the result of interspecific competition [108]. As no invasive necrophagous fly species such as *Ch. rufifacies* and *L. cuprina* have been recorded to date in New Haven, this is an example of a native biota that has been exposed to persistent disturbance and is experiencing biotic homogenization driven by the hyper-proliferation of native species rather than being replaced by alien species [107].

Urbanization can result in an increase in total insect abundance due to a higher abundance of generalist urbanophiles and the concentration of resources [109]. Arthropods in most urban systems are controlled through resource-based competition or bottom-up forces [110]. While the availability of domestic food waste and animal feces increases the availability of food, the poor nutritional value of such substances is often unable to sustain developing larvae, reducing survival, fecundity, and the size of adults [111,112]. While such an observation is anecdotal at this stage, *L. sericata* adult specimens in the current survey were noticeably smaller than specimens examined from other regions. *Phormia regina*, one of the world’s most common species, may be an example of a species that has been lost from a region, with historical materials suggesting the species may have been present once, but it is now extinct in Britain and Sardinia [113].

Several differences should be noted between the trapping methodology used in the 1940s and the present day survey. Not all of the specimens trapped in the 1940s survey were identified; rather, 100 flies were randomly selected and identified from each trap. Fly numbers for the 1940s survey were also expressed in volume (cm^3^), while in the current survey, all specimens were counted and identified. Given that not all of the specimens in the 1940s survey were identified, the difference in species diversity between the two surveys may be greater. The number of trapped flies was far greater in the 1940s, and some of this difference may be accounted for by the presence of a permanent source of horse manure and bi-weekly trappings rather than weekly. However, equally large numbers of flies were trapped weekly in a 1959–1960 New Haven study, where no horse manure was present, and a bait of similar size and type (chicken intestines) was used [80]. The number of flies entering a trap in the current survey never reached the abundance and diversity of the 1940s survey, despite baiting one of the present day traps with a decomposing pig head on a 25 degrees day in the early fall.

As noted, different type of fly traps were used in the 1940s and the current survey; however, they are of a similar type and size. The hanging Diptera cone trap [42] used in the present day study and the conical hoop flytrap [1,43] used in the 1940s survey are of similar design, composed of an inverted screen cone inside a screen cylinder placed over a container holding bait. In both traps, flies enter the elevated base to access the bait and are trapped when flying or walking upwards through a small hole at the apex of the cone and into the cylinder. Both traps work on the principle that flies will fly upwards towards light after having been attracted beneath [43]. Both traps used had a base measuring approximately 30 cm wide and a cylinder approximately 30 cm high.

Different baits were used in the 1940s and the present-day survey: pooled mouse tissues and feces versus raw pork, while of comparable size. Past research has shown that the addition of manure to meat does increase the bait’s attractiveness for some *Calliphora* and *Chrysomya* species, but not for *Lucilia sericata* [114]; the type of animal meat does not influence species richness or abundance [115], and *L. sericata, L. coeruleiviridis* and *P. regina* were equally attracted to beef liver and whole piglet carcasses [81]. However, the absence of manure from the present-day trap may explain why some species were not trapped, for example, species of the *Scathophaga* genus, which are primarily attracted to manure. The absence of manure from the present-day trap may account for the absence of four of the species listed in Table 4: *Ch. splendida, D. picta, Toxomerus geminatus*, and *Scathophaga* spp, while all other species have been shown to colonize carrion or frequent carrion baited traps [42,81,116,117,118,119,120].

The key difference between the trapping methodologies was trap placement. While the traps in both studies were placed between the hours of 9 a.m. and 4 p.m., the trap in the present day survey was hung approximately 1.5 m from the ground, while the trap in the 1940s survey would have been placed on the ground. *Lucilia sericata* trap numbers have been shown to be unaffected when comparing trap heights of 31, 61, and 91 cm [121]. A trap height of 0.5 m, compared to above 1 m, has been shown to affect numbers of *L. cuprina*, with a lower mean catch at 0.5 m [122]; however, such results suggest the low height of the 1940s trap may have limited trap numbers. Without data comparing the attractiveness of the two traps and baits used, it is difficult to rule out any influence these variables may have had on the number and diversity of flies trapped. However, given the similarity in methodology, it is believed that this influence would have been minimal compared to the long-term effects of changes in habitat and climatic variables.

Fly populations fluctuated markedly from year to year in the current survey and in the 1940s survey. *Lucilia. sericata, P. regina*, and *L. coeruleiviridis* populations fluctuated markedly from year to year in the current survey, and *L. sericata, P. regina*, and *L. illustris* populations fluctuated from year to year in the 1940s survey. Volatile population dynamics are common in flies and other insects due to their ectothermic nature [123]. The development and oogenesis of flies is closely linked to temperature and precipitation, resulting in the synchronism of numerous generations and a quick increase in fly populations [124]. Such fluctuations make the interpretation of species-level demographic data difficult, as an annual decline of 1% to 2% is difficult to discern in the midst of such heterogeneity [30]. To determine if a decline or change is substantial and permanent, data should ideally be collected over several decades or include aggregate data at higher taxonomic levels [30]. The current survey period was limited to two years, and the comparative periods are separated by a wide gap in time rather than a continuous record. This makes comparative analysis less than ideal, but still valid. The results of this study show a simultaneous change in several Diptera families and are complemented by survey data collected in the same and nearby states. In addition, a population variation characteristic of calliphorids and other families trapped would have been captured in survey data given that both surveys were conducted over several weeks and over multiple years. Local-scale studies are necessary as they can highlight changes in populations that are often lost in multitaxon studies that combine insects from different regions with different ecological needs [30]. While the global decline of insects is evident, patterns of variation exist at the local scale, necessitating local surveys such as this to help identify local-scale drivers responsible for changes in population trends [28].

It was fortuitous in one sense that a survey of the local fly population in New Haven, Connecticut, was made in the 1940s due to questions regarding whether flies transmit polio after the virus was isolated from several fly species. Ten years later, in the early 1950s, the transmission of polio by flies was no longer viewed as important after fly abatement efforts demonstrated that flies were not a true host of the virus and any fly transmission was considered incidental [125,126,127]. However, fly surveys were repeated in New Haven in 1959–1960 and concluded that polio was readily found in flies during an urban epidemic. The presence of the virus in flies mirrored the level of clinical disease in the population, and flies were considered to be a possible source of epidemic variant strains [80,128].

Today, the role flies play in an epidemic is again being questioned with the current novel severe acute respiratory syndrome coronavirus 2 (SARS-CoV-2) pandemic. SARS-CoV-2 was first detected in New Haven in March 2020, nine months after the current fly survey began. While the World Health Organization considers the flies’ role in the transmission of the virus to again be incidental [129] research is starting to emerge demonstrating that flies are capable of transmitting SARS-CoV-2 and that infected flies are present in field samples from infected communities [130,131]. As the pandemic continues across the US and the world, an increase in fly surveys should be anticipated, providing the impetus to generate further comparative studies of domestic filth flies.

## Figures and Tables

**Table 1:** Fly species trapped in the current 2019–2020 survey.

2019–2020 Traps					
Species	Position Based on Sum of Arbitrary Ranks	Sum of Arbitrary Ranks	Count Rank	Sum of Total Specimen Counts	Percent of Overall Count
*Lucilia sericata*	1	325	1	764	56.76
*Lucilia coeruleiviridis*	2	317	2	342	25.41
*Phormia regina*	3	208	3	180	13.37
*Muscina stabulans*	4	70	5	9	0.67
*Hylemya* spp.	5	61	4	13	0.97
*Calliphora vicina*	6	60	7	6	0.45
*Hydrotaea ignava*	7	48	6	8	0.59
*Muscina assimilis*	8	41	8	4	0.30
*Calliphora livida*	9	30	6	8	0.59
*Lucilia silvarum*	10	29	8	4	0.30
*Sarcophaga* spp.	11	28	9	3	0.22
*Fannia* spp.	12	20	8	4	0.30
*Musca domestica*	13	10	10	1	0.07

**Table 2:** Fly species trapped in the 1943–1944 survey.

1943–1944 Traps					
Species	Position Based on Sum of Arbitrary Ranks	Sum of Arbitrary Ranks	Volume Rank	Sum of Volume in cc	Percent of Overall Count
*Lucilia sericata*	1	803	1	21,698	83.75
*Phormia regina*	2	593	3	1344	5.19
*Lucilia illustris*	3	463	2	1937	7.48
*Sarcophaga* spp.	4	347	4	304	1.17
*Calliphora vicina*	5	187	13	36	0.14
*Lucilia coeruleiviridis*	6	177	7	100	0.39
*Hylemya* spp.	7	155	5	102	0.39
*Cynomyopsis cadaverina*	8	151	9	68	0.26
*Muscina stabulans*	9	143	8	75	0.29
*Fannia* spp.	10	121	6	101	0.39
*Musca domestica*	11	103	11	49	0.19
*Calliphora vomitoria*	12	83	12	37	0.14
*Hydrotaea ignava*	13	70	10	58	0.22

**Table 3:** Comparison of fly species trapped in the 1940s and current survey. Species with a significantly different fly count between surveys are in bold.

Species	1943–1944 Traps			2019–2020 Traps			Difference	
	Arbitrary Rank	Volume Rank	Percent of Overall Count	Arbitrary Rank	Count Rank	Percent of Overall Count	z Score	*p* Value
***L. sericata***	1	1	83.75	1	1	56.76	−25.36	**<0.0001**
***P. regina***	2	3	5.19	3	3	13.37	12.73	**<0.0001**
***L. illustris***	3	2	7.48	0	0	0	−10.41	**<0.0001**
***Sarcophaga* spp.**	4	4	1.17	11	9	0.22	−3.22	**<0.001**
***C. vicina***	5	13	0.14	6	7	0.45	2.82	**<0.01**
***L. coeruleiviridis***	6	7	0.39	2	2	25.41	70.77	**<0.0001**
***Hylemya* spp.**	7	5	0.39	5	4	0.97	3.21	**<0.001**
*Cy. cadaverina*	8	9	0.26	0	0	0	−1.87	Not sig
*M. stabulans*	9	8	0.29	4	5	0.67	2.45	Not sig
*Fannia* spp.	10	6	0.39	12	8	0.30	−0.52	Not sig
*M. domestica*	11	11	0.19	13	10	0.07	−1.00	Not sig
*C. vomitoria*	12	12	0.14	0	0	0	−1.37	Not sig
***H. ignava***	13	10	0.22	7	6	0.59	2.71	**<0.01**
***C. livida***	0	0	0	10	6	0.59	12.37	**<0.0001**
*M. assimilis*	14	14	Unknown *	8	8	0.30	n/a	n/a
*L. silvarum*	14	14	Unknown *	9	8	0.30	n/a	n/a

* Percentages not provided in 1940s publication.

**Table 4:** Differences in fly species trapped in the current and 1940s surveys.

Species Found in Both the 1940s Traps and the 2019–2020 Traps	Species Found in the 1940s Traps but not in the 2019–2020 Traps	Species Found in 2019–2020 Traps but not in 1940s Traps
*Lucilia sericata* *Phormia regina* *Lucilia coeruleiviridis* *Lucilia silvarum* *Calliphora vicina* *Sarcophaga *spp. *Muscina stabulons* *Hylemya* spp. *Fannia* spp. *Muscina domestica* *Muscina assimilis* *Hydrotaea ignava*	*Lucilia illustris †* *Calliphora vomitoria †* *Cochliomyia macellaria* *Cynomyopsis cadaverina* *Protophormia terraenovae* *Pollenia rudis †* *Helina* spp. *Myospila meditabunda* *Scathophaga* spp. *Stomoxys calcitrans* *Chrysomyza splendida* *Euxesta notata* *Delphinia picta* *Ptecticus trivitattus* *Syritta pipiens* *Toxomerus geminatus*	*Calliphora livida*

† trapped in Hartford County in same time period.

## Data Availability

No data availability.
